# Angiotensin II upregulates RANKL/NFATC1 expression in synovial cells from patients with rheumatoid arthritis through the ERK1/2 and JNK pathways

**DOI:** 10.1186/s13018-021-02451-0

**Published:** 2021-05-05

**Authors:** Zhiping Zhao, Yongtao Zhang, Changyao Wang, Xiangyu Wang, Yingzhen Wang, Haining Zhang

**Affiliations:** 1 Department of Joint Surgery, The Affiliated Hospital of Qingdao University, Qingdao, 266000 Shandong China; 2 Department of Orthopedics, The Affiliated Hospital of Qingdao University, 59 Hai Er Road, Qingdao, Shandong 266061 P.R. China

**Keywords:** Angiotensin II, RANKL, NFATC1, Synovial cells, ERK1/2, JNK pathways

## Abstract

**Background:**

Angiotensin II (Ang II) is associated with rheumatoid arthritis (RA) development. The present study investigated the impact of Ang II on the expression of receptor activator of nuclear factor-κB ligand (RANKL), as well as of nuclear factor of activated T cells cytoplasmic 1 (NFATC1) in RA synovial cells, and explored the underlying mechanism.

**Methods:**

The expression levels of RANKL, NFATC1, and Ang II type 1 receptor (AT1R) were analyzed by RT PCR, western-blot, and/or immunohistochemistry. Western blot was also used to analyze the p38MAPK, JNK, and ERK1/2 pathways.

**Results:**

The expressions of RANKL and NFATC1 increased in synovial tissues of RA compared to osteoarthritis (OA) synovial tissues. The expression of RANKL was upregulated by Ang II, and this effect was mitigated by an AT1R blocker but not by an AT2R blocker. Furthermore, Ang II activated the ERK1/2, JNK, and p38MAPK pathways, and this effect was blocked by the AT1R blocker. However, ERK1/2 and JNK inhibitors, but not a p38MAPK inhibitor, blocked Ang II-induced RANKL expression. Ang II also increased the level of NFATC1, and this upregulation was attenuated by AT1R blockade, ERK1/2 and JNK inhibition, and siRNA-mediated RANKL silencing, but not by AT2R blockade or p38MAPK inhibition.

**Conclusion:**

Our results indicated that Ang II activated the ERK1/2 and JNK pathways via AT1R, thus upregulating RANKL and NFATC1 expressions in RA synovial cells.

## Highlights

1. RANKL and NFATC1 expressions increase in RA synovial tissues compared with in OA synovial tissues.
2. Ang II upregulates the expressions of RANKL and NFATC1 in synovial cells.
3. Ang II induces RANKL production via the AT1R/ERK/JNK pathway.
4. Ang II induces NFATC1 production via the AT1R/ERK/JNK/RANKL pathway.

## Introduction

Rheumatoid arthritis (RA) is characterized by progressive destruction of periarticular bone, mainly mediated by osteoclasts, a process that is highly associated with joint deformity and patient disability [1–3]. Receptor activator of nuclear factor κB ligand (RANKL) is the most important inducer of osteoclastogenesis [4–6]. Many studies have shown that RANKL is expressed and upregulated in the synovial tissues of RA patients and animal models, and that increased RANKL expression in synovial cells is responsible for osteoclastogenesis during RA [3, 7–9]. For example, Shigeyama et al. [8] showed that RANKL is expressed in synovial cells and that synovial cells with high RANKL expression are strong inducers of osteoclast-like cells. Danks et al. [3] reported that RANKL in synovial cells is implicated in osteoclast formation and bone erosion in RA mice. We have previously shown that the RANKL level is higher in the synovial tissues of RA rats compared to normal rats [10]. However, although previous studies have reported that many proinflammatory cytokines, such as interleukin (IL)-18 [11], IL-6 [12], IL-22 [13], and IL-29 [14], may induce RANKL expression in fibroblast-like synoviocytes, the underlying mechanism regulating RANKL expression in RA synovial cells has not been elucidated.

The renin-angiotensin system (RAS), a well-known hormone system that regulates blood pressure, as well as fluid and electrolyte homeostasis, has been identified in bone tissues, and participates in the regulation of bone metabolism [15–18]. For example, we previously showed that local RAS in bone tissues is involved in the pathogenesis of glucocorticoid-induced osteoporosis via the stimulation of bone resorption and the inhibition of bone formation [18]. Hatton et al. [16] reported that Angiotensin II (Ang II), a primary effector in RAS, exhibits a potent effect on osteoclastogenesis. However, it was also demonstrated that Ang II indirectly promotes osteoclast differentiation and activation by inducing RANKL upregulation in osteoblasts, rather than directly acting on osteoclasts [15, 17]. Similarly, the local RAS also expressed in synovial tissues, and treatment with angiotensin-converting enzyme (ACE) inhibitors or angiotensin type 1 receptor (AT1R) blocker mitigated RA symptoms in animal models [19–23]. Moreover, we recently found that activated RAS increases RANKL expression in synovial tissues and induces bone resorption [10]. However, to date, the impact of Ang II on the expression of RANKL in isolated synovial cells is unknown.

Mitogen-activated protein kinases (MAPKs), comprising several subfamilies, including p38MAPK, c-jun NH2-terminal kinase (JNK), and extracellular signal-regulated kinase (ERK) 1/2, are activated by Ang II and participate in various disease processes [23–25]. The study of Lu et al. [25] showed that Ang II induces a dose-dependent increase in the levels of phosphorylated ERK1/2, JNK, and p38 MAPK without affecting the total level of these proteins in human aortic smooth muscle cells. Hu et al. [24] found that Ang II induces cardiac hypertrophy in mice, by activating the ERK1/2, JNK, and p38 MAPK pathways in myocardial tissues. Another study indicated that AT1R blockade mitigates the inflammatory response by inhibiting the ERK and p38 MAPK pathways in both T and B cells of RA rats [23]. Moreover, the activation of ERK1/2, JNK, and/or p38 MAPK induced by IL-29, IL-6, CXC-motif ligand (CXCL) 16, and tumor necrosis factor (TNF)-α increases the expression of RANKL in fibroblast-like synoviocytes [12, 14, 26, 27]. Thus, Ang II-activated MAPK signaling leads to increased RANKL expression in synovial cells.

Nuclear factor of activated T cells, cytoplasmic 1 (NFATC1) is a crucial transcription factor for osteoclastogenesis, and NFATC1could regulated osteoclast activation factors such as TRAP and cathepsin K [6]. RANKL induces osteoclastogenesis mainly by binding to the receptor RANK of osteoclast precursors and then activates NFATC1 [28]. Previous studies have shown that increased NFATC1 expression in bone marrow macrophages induces osteoclastogenesis, while NFATC1-deficient cells do not differentiate into osteoclasts, even after stimulation with RANKL [29, 30]. Therefore, we hypothesized that Ang II may induce RANKL expression and increase NFATC1 level via the MAPK pathway in human RA synovial cells. In the present study, human RA synovial cells were used to explore the effects of Ang II on the expressions of RANKL and NFATC1, as well as the underlying mechanism.

## Materials and methods

### Patients and tissue specimens

Synovial tissues were obtained from 3 patients with RA and 3 patients with osteoarthritis (OA) undergoing knee replacement surgery. RA diagnosis was made according to the American College of Rheumatology 1987 revised criteria [31], while OA patients were diagnosed based on accepted diagnostic criteria [32]. The clinical characteristics of patients are shown in Table 1. This study was approved by the Ethical Committee of the Affiliated Hospital of Qingdao University, China, and an informed consent was obtained from all patients. Part of the synovial tissues were frozen and stored at −80 °C for further analysis. The remaining tissues were fixed in 10% neutral buffered formalin and embedded in paraffin.

**Table 1:** Clinical characteristic of the patients with rheumatoid arthritis

Patient	Gender	Age (years)	Site of surgery	RA duration (months)
RA 1	Female	62	Knee	240
RA 2	Female	58	Knee	180
RA 3	Male	66	Knee	300
OA 1	Female	65	Knee	/
OA 2	Female	61	Knee	/
OA 3	Male	63	Knee	/

### Immunohistochemistry

Sections (4 μm) of synovial tissue from RA and OA patients were prepared. After a regular deparaffinization procedure, the sections were incubated with 0.3% hydrogen peroxidase for 15 min at room temperature. Subsequently, antigen retrieval was obtained by using 0.01 M citrate buffer at 80 °C for 20 min. Next, the sections were blocked with normal goat serum for 30 min at room temperature, and incubated with primary antibodies against RANKL (Abcam, USA, 1:500) or NFATC1 (Santa Cruz Biotechnology; 1:200). Finally, the sections were incubated with biotinylated secondary antibody (Zhongshan Golabrige Biotechnology, China) followed by horseradish peroxidase-conjugated streptavidin (Zhongshan Golabrige Biotechnology, China). The signals were visualized by diaminobenzidine, captured by a microscope (Leica Microsystems, Wetzlar, Germany), and photographed.

### Cell primary culture and treatment

Synovial cells were isolated from synovial tissues of RA patients undergoing knee replacement surgery according to a previously described method [9]. Briefly, synovial tissues were washed with RPMI 1640 (Gibco BRL, USA), minced, and digested with collagenase (Sigma, USA) and DNase I (Sigma, USA) at 37 °C for 90 min, with shaking. The cell suspension was filtered using a 70-μm cell strainer, layered on a Ficoll/Paque density gradient, and centrifuged at 400 g for 30 min at room temperature. The interface layer was resuspended in RPMI 1640 and centrifuged at 250 g for 10 min. Finally, the cells were cultured in minimum essential medium (a-MEM; Gibco BRL, USA) containing 20% horse serum (Sigma, USA) which had been heated at 56 °C for 30 min. Synovial cells from passages 3–8 in culture were collected and used in the next experiment. Synovial cells from each RA patient were used separately in three independent experiments for each of the assays described below. In the following experiments, synovial cells were treated in the absence or presence of Ang II (10^−10^ M-10^−6^ M), AT1R blocker (Olmesartan, 10^−5^ M), AT2R blocker (PD12331910, 10^−5^ M), MEK1/2 inhibitor (U0126, 5×10^−6^ M), p38MAPK inhibitor (SB203580, 1×10^−5^ M), or JNK inhibitor (SP600125, 1×10^−5^ M), and then harvested for subsequent analyses.

### Cell Counting Kit-8 (CCK8) assay

Synovial cells were seeded in 96-well plates and treated with vehicle control or different concentrations of Ang II for 48 h. The viability of synovial cells was determined by a CCK-8 kit (Dojindo Laboratories, China) according to the manufacturer’s protocol.

### Western blot

Tissues and cells were washed with ice-cold PBS and then homogenized in RIPA Lysis Buffer (Beijing Solarbio Science & Technology, China) containing 1% protease inhibitor (MedChemExpress) and 1% phosphatase inhibitor (MedChemExpress). The samples were centrifuged after 30 min of incubation on ice. The protein concentration was determined by a bicinchoninic acid (BCA) protein assay kit (Beijing Solarbio Science & Technology, China). Protein samples combined with loading buffer were heated to 95 °C for 5 min. Next, equivalent amounts of protein samples (15 μl) were loaded onto SDS gels, and electrophoresis was conducted for 120 min at a constant voltage of 90 V, and then transferred onto PVDF membranes. The membranes were blocked with 5% fat-free milk TBS-Tween buffer for 2 h at room temperature. The proteins were detected by using anti-RANKL (Abcam, USA, 1:1000), anti-NFATC1 (Santa Cruz Biotechnology; 1:1000), anti-AT1R (Abcam, USA, 1:800), anti-p38MAPK (Cell Signaling Technology, USA, 1:1000), anti-phospho-p38MAPK (Cell Signaling Technology, USA, 1:1000), anti-JNK (Cell Signaling Technology, USA, 1:1000), anti-phospho-JNK (Cell Signaling Technology, USA, 1:1000), anti-ERK1/2 (Cell Signaling Technology, USA, 1:1000), anti-phospho-ERK1/2 (Cell Signaling Technology, USA, 1:1000), and anti-GAPDH (Cell Signaling Technology, USA, 1:2000) antibodies, overnight at 4 °C. Membranes were washed and incubated with HRP-conjugated secondary antibodies (Cell Signaling Technology, USA, 1:3000). The target bands were visualized using ECL-PLUS reagents (Sigma, USA), and were scanned using a BioSpectrum Imaging System (UVP, Thermo Fisher Scientific, USA). The results of western blot analysis were quantified through integrated density using the Image Pro Plus 6.0 software and were normalized to GAPDH levels.

### Real-time polymerase chain reaction (PCR)

Total RNA was extracted from synovial cells or human tissue samples using TRIzol reagent (Invitrogen Life Technologies, USA) according to the manufacturer’s protocol, and the RNA was transcribed into cDNA using the First Strand cDNA kit (Fermentas, Canada). Next, we used the SYBR Green PCR master mix (TaKaRa, Japan) to evaluate the mRNA expressions of the target genes by real-time PCR in an ABI 7300 RTPCR system. Human GAPDH was used as a reference gene and the relative expressions of the target genes were analyzed by the 2^−ΔΔCt^ method. All primer sequences are shown in Table 2.

**Table 2:** Sequences of primers used

Target genes	Primer sequences (5′-3′)	GenBank no.
RANKL	F: ACTACACCAAGTACCTGCGT	NM_003701.4
	R: TAGGATCCATCTGCGCTCTG	
AT1R	F: TCCAAGATGATTGTCCCA	NM_032049.3
	R: CTATCACCACCAAGCTGT	
NFATC1	F: GCATCACAGGGAAGACCGTGTC	NM_001278675.2
	R: GAAGTTCAATGTCGGAGTTTCTGAG	
GAPDH	F: AACGGGAAGCTTGTCATC	NM_001357943.2
	R: ACTCCACGACGTACTCAG	

### Transfection of small interfering RNA (siRNA) into RA synovial cells

RA synovial cells were seeded in 6-well plates (5×10^4^ cells per well) and incubated overnight. Then, control siRNA (si-Ctr) or RANKL-specific siRNA (siRNA-RANKL) (Santa Cruz, USA) were transfected by using siRNA Transfection Reagent (Santa Cruz, USA) according to the manufacturer’s instructions. The efficacy of RANKL silencing was assessed by real-time PCR.

### Statistical analysis

Data were expressed as mean ± standard deviation (SD). Differences between groups were evaluated by one-way analysis of variance followed by LSD *t* test for subgroup analysis (SPSS 17.0 SPSS Inc., Chicago, IL, USA), and *P* values < 0.05 were considered statistically significant.

## Results

### The expressions of RANKL and NFATC1 increased in the RA synovial tissues compared with that in the OA synovial tissues

**Fig. 1:**
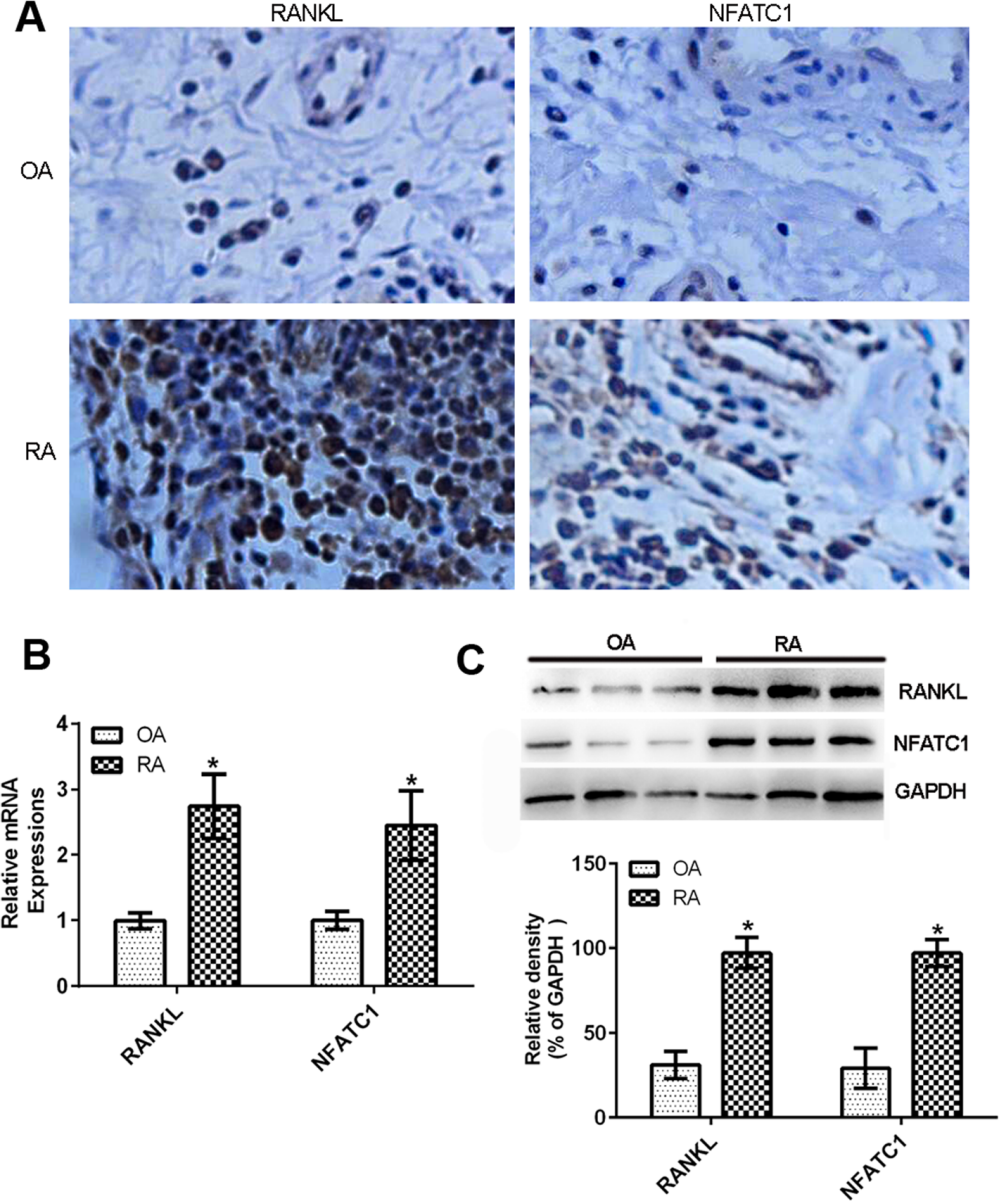
Upregulated RANKL and NFATC1 expressions in the synovial tissues of RA patients compared with that of OA patients. The expressions of RANKL and NFATC1 in synovial tissues from RA patients and OA patients were analyzed by immunohistochemistry, RT-PCR, and western blot. Data are representative images or expressed as the mean ± SD of each group from 3 separate patients. **a** Representative immunohistochemistry images (magnification ×400) (*n* = 3). **b** RT-PCR analysis of RANKL and NFATC1 mRNA levels (*n* = 3). **c** Western blot detection of RANKL and NFATC1 (*n* = 3). *p < 0.05 vs the OA group

The expressions of RANKL and NFATC1 were examined by immunohistochemistry in RA synovial tissues of patients with RA or OA. The results showed that RANKL and NFATC1 expressions were higher in the synovial tissues of RA patients than in those of OA patients (Fig. 1a). In addition, real-time PCR confirmed that of RANKL and NFATC1 mRNA levels were significantly higher in the synovial tissues of RA patients compared to those of OA patients (Fig. 1b). Consistently, RANKL and NFATC1 proteins were more abundant in the synovial tissues of RA patients than in those of OA patients (Fig. 1c).

### Ang II-induced RANKL expression in RA synovial cells

**Fig. 2:**
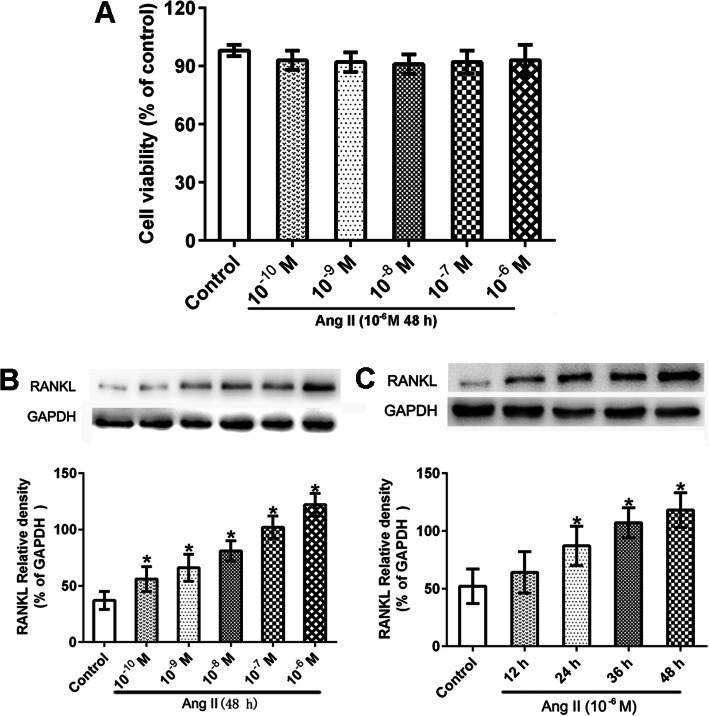
Ang II increased RANKL expression in synovial cells. Synovial cells were cultured in triplicate in the presence or absence of Ang II for the indicated times. Data are representative images or expressed as the mean ± SD of three independent experiments with synovial cells from each RA patient. **a** Viability of cells exposed to different concentrations of Ang II was determined by CCK8 assay (*n* = 3). **b** Western blot analysis of RANKL expression in cells exposed to different concentrations of Ang II (*n* = 3). **c** Western blot analysis of RANKL expression at different times (*n* = 3). *p < 0.05 vs. the control group

Our previous study demonstrated that RAS activation in the synovial tissues promotes osteoclastogenesis via the RANKL/RANK pathway [10]. Therefore, we sought to determine whether Ang II could stimulate RANKL expression in RA synovial cells. The viability of RA synovial cells was determined by a CCK8 assay, showing that a 48-h incubation with Ang II had no effect on cell viability at any of the employed concentrations (10^−10^ M-10^−6^ M) (Fig. 2a). Then, the impact of the latter experimental conditions on the protein level of RANKL was determined by western blot in RA synovial cells. Ang II increased the protein level of RANKL in RA synovial cells in a dose-dependent manner (Fig. 2b). Furthermore, when RA synovial cells were treated with 10^−6^ M Ang II, RANKL protein level increased in a time-dependent manner (Fig. 2c).

### Ang II-induced RANKL expression in RA synovial cells via AT1R

**Fig. 3:**
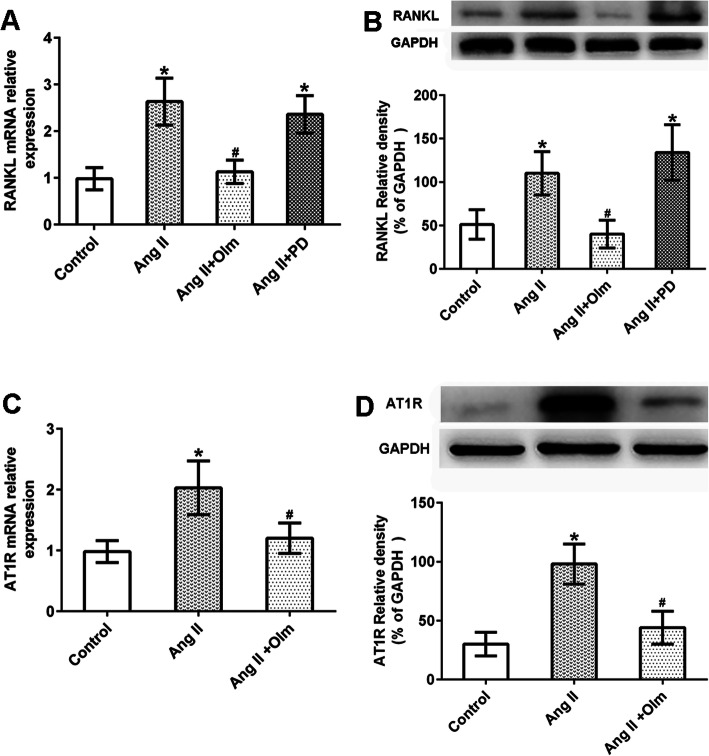
Ang II increased RANKL expression in synovial cells via AT1R. Synovial cells were cultured in triplicate with or without the AT1R antagonist (olmesartan, Olm, 10^−5^ M) or the AT2R antagonist (PD123319, PD, 10^−5^ M) for 30 min, and exposed to Ang II (10^−6^ M) for 48 h. The relative level of RANKL was determined by RT-PCR and western blot. Data are representative images or expressed as the mean ± SD of three independent experiments with synovial cells from each RA patient. **a** RT-PCR analysis of AT1R expression (*n* = 3). **b** Western blot analysis of AT1R expression (*n* = 3). **c** RT-PCR analysis of AT1R expression (*n* = 3). **d** Western blot analysis of AT1R expression (*n* = 3). *p < 0.05 vs. the control group; ^#^*p* < 0.05 vs. the Ang II group

It is known that Ang II exerts its biological effects by binding to AT1R and AT2R. Therefore, we attempted to identify the receptor involved in Ang II-induced RANKL expression. Notably, the effect of Ang II on RANKL mRNA and protein expressions were prevented by cell pre-treatment with olmesartan but not with PD123319, which suggested that Ang II increased the expression of RANKL via AT1R, rather than AT2R (Fig. 3a and b).

To determine the effect of Ang II on AT1R expression, RA synovial cells were pretreated with or without olmesartan, and then exposed to Ang II (10^−6^ M) for 48 h. As shown in Fig. 3c, treatment with Ang II significantly increased the mRNA level of AT1R, and this effect was reversed by treatment with olmesartan. A similar pattern for the protein level of AT1R was detected under different treatment conditions in RA synovial cells (Fig. 3d).

### Ang II-induced RANKL expression in RA synovial cells was mediated by ERK1/2 and JNK signaling

**Fig. 4:**
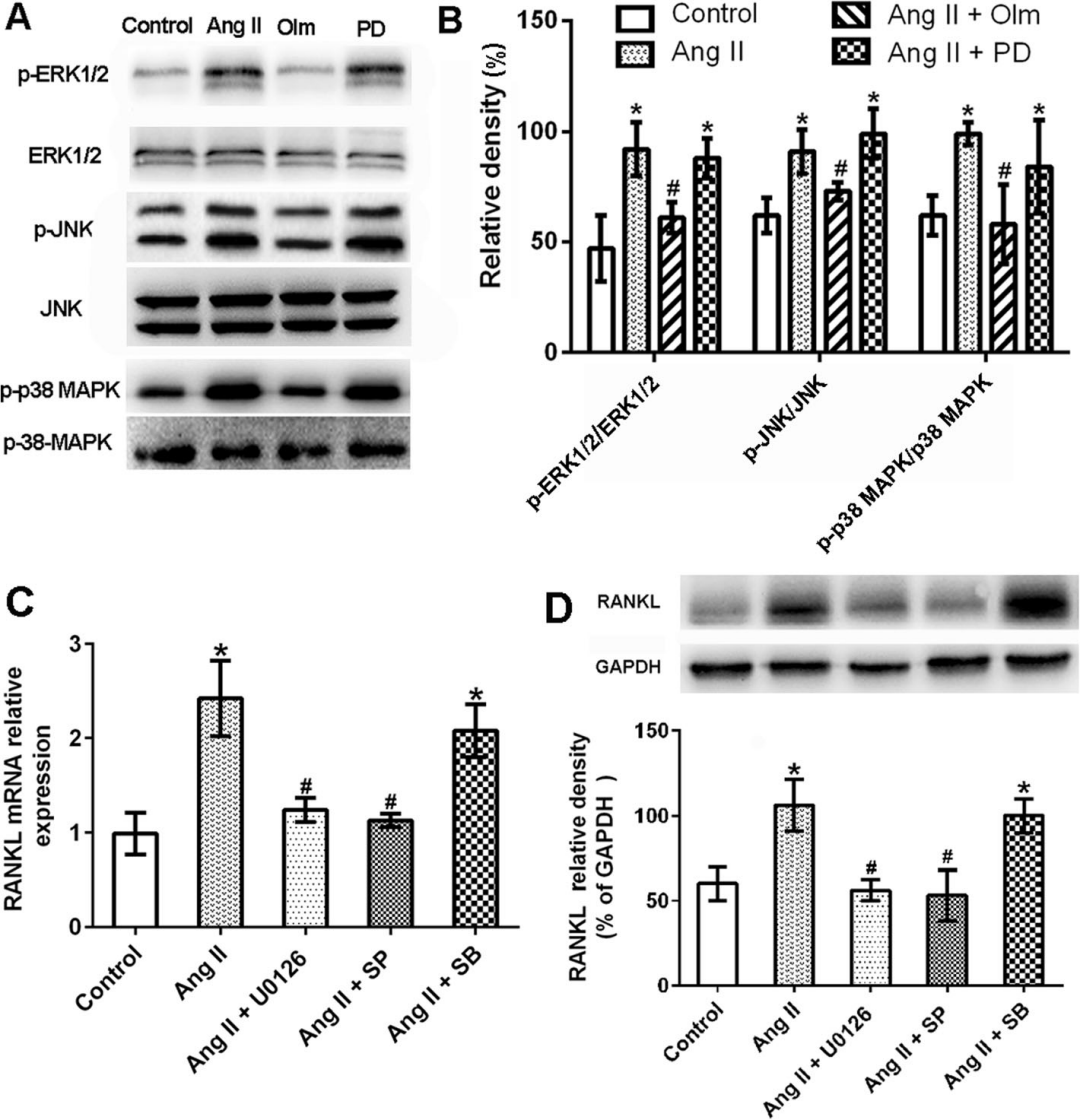
Ang II increased RANKL expression in synovial cells via the ERK1/2 and JNK pathways. Synovial cells were pretreated in triplicate with or without the AT1R antagonist (olmesartan, Olm, 10^−5^ M) or AT2R antagonist (PD123319, PD, 10^−5^ M) for 30 min, and then exposed to Ang II (10^−6^ M) for 48 h. The relative levels of ERK1/2, JNK, and p38MAPK phosphorylation were characterized by western blot. Synovial cells were pretreated in triplicate with or without the ERK1/2 inhibitor (U0126, 5×10^−6^ M), the JNK inhibitor (SP600125, SP 1×10^−5^ M) or the p38MAPK inhibitor (SB203580, SB, 1×10^−5^ M) for 30 min, and then exposed to Ang II (10^−6^ M) for 48 h. The relative level of RANKL was determined by RT-PCR and western blot. Data are representative images or expressed as the mean ± SD of three independent experiments with synovial cells from each RA patient. **a** and **b** Western blot analysis of ERK1/2, JNK, and p38MAPK phosphorylation level (*n* = 3). **c** RT-PCR analysis of RANKL expression (*n* = 3). **d** Western blot analysis of RANKL expression (*n* = 3). *p < 0.05 vs. the control group; ^#^*p* < 0.01 vs. the Ang II group

To clarify the molecular mechanism underlying the effect of Ang II on RANKL expression, the phosphorylation of MAPKs, i.e., ERK, JNK, and p38 MAPK was evaluated by western blot. The results showed that the phosphorylation of these proteins rapidly increased in RA synovial cells after treatment with Ang II, and that such effect was blocked by pretreatment with olmesartan, but not with PD123329 (Fig. 4a and b). Next, the effect of ERK, JNK, and p38 MAPK inhibitors on RA synovial cells was explored. In particular, U0126 and SP600125 attenuated Ang II-induced upregulation of RANKL, whereas SB203580 had no effect (Fig. 4c and d). These results suggested that the ERK1/2 and JNK pathways were important for Ang II-induced RANKL upregulation in RA synovial cells.

### NFATC1 was induced by Ang II in RA synovial cells

**Fig. 5:**
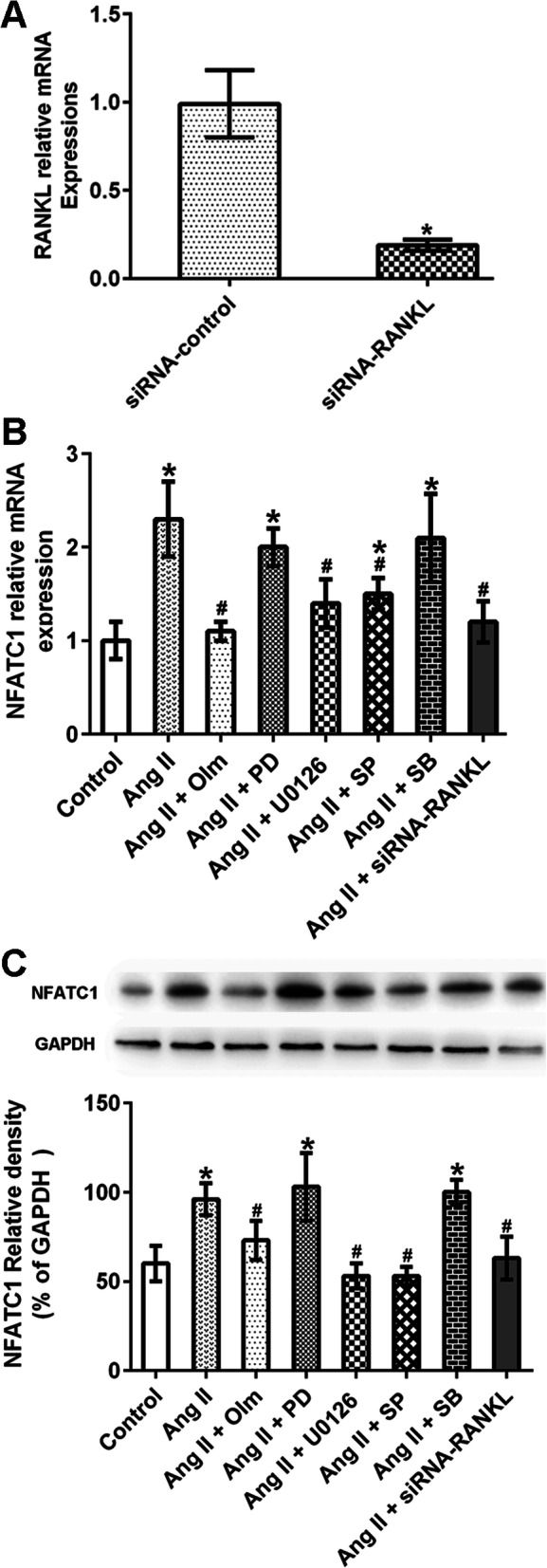
Ang II increased NFATC1 expression in synovial cells via the RANKL pathway. Synovial cells were pretreated with or without the AT1R antagonist (olmesartan, Olm, 10^−5^ M), the AT2R antagonist (PD123319, PD, 10^−5^ M), the ERK1/2 inhibitor (U0126, 5×10^−6^ M), the JNK inhibitor (SP600125, SP 1×10^−5^ M), or the p38MAPK inhibitor (SB203580, SB, 10^−5^ M) for 30 min, or transfected with control or RANKL-specific siRNA for 48 h, and then exposed to Ang II (10^−6^ M) for 48 h. The relative mRNA and protein levels of RANKL and NFATC1 in different groups were evaluated by RT-PCR and western-blot. Data are representative images or expressed as the mean ± SD of three independent experiments with synovial cells from each RA patient. **a** The relative level of RANKL mRNA transcripts was determined by RT-PCR (*n* = 3). **b** RT-PCR analysis of NFATC1 expression (*n* = 3). **c** Western blot analysis of NFATC1 expression (*n* = 3). *p < 0.01 vs. the control group; ^#^*p* < 0.01 vs. the Ang II group

To characterize the effect of Ang II on NFATC1 expression and the role of RANKL in such effect, synovial cells were pretreated with various signaling inhibitors, as well as siRNA-RANKL transfection. First, we verified the efficiency of siRNA-mediated RANKL silencing by using real-time PCR, and found that the transfection with siRNA-RANKL reduced RANKL expression in RA synovial cells (Fig. 5a). Next, NFATC1 expression was analyzed by real-time PCR and western blot. The results showed that olmesartan, U0126, SP600125, and siRNA-RANKL attenuated Ang II-induced NFATC1 upregulation, whereas PD123329 and SB203580 had no effects (Fig. 5b and c). These results indicated that Ang II promoted the expression of RANKL via the ERK1/2 and JNK pathways, ultimately upregulating NFATC1 expression.

## Discussion

The present study demonstrated that the expressions of RANKL and NFATC1 in synovial tissues were higher in patients with RA than in OA patients. The mechanism underlying this phenomenon was explored, and the results showed that Ang II-induced RANKL expression required AT1R-mediated ERK1/2 and JNK pathway activation. Moreover, we investigated the effect of Ang II on the expression of NFATC1, which is a central cytokine in RANKL-induced osteoclastogenesis in RA synovial cells, as well as the responsible mechanism. Ang II-induced NFATC1 expression required AT1R, as well as the ERK1/2, JNK, and RANKL pathways. These data demonstrated that, in RA synovial cells, Ang II enhanced RANKL production via AT1R, as well as ERK1/2 and JNK activation, ultimately causing NFATC1 upregulation.

Previous studies reported RANKL overexpression in RA synovial tissues [8, 9]. Recently, Li et al. [27] found that RANKL expression in synovial tissues was upregulated in RA patients compared to OA patients, in line with the present study. NFATC1, the main cytokine involved in RANKL-induced osteoclastogenesis, was also upregulated in active RA synovial tissues, compared to inactive RA, OA, or normal synovial tissues [33] and our results confirmed that NFATC1 was expressed in synovial tissues, and that its level was higher in RA patients compared to OA patients.

RAS is associated with bone metabolism and Ang II is a potent stimulator of osteoclastogenesis [16, 34]. However, Ang II does not target osteoclasts directly, and many studies have clearly demonstrated that Ang II promotes osteoclastogenesis by inducing the expression of RANKL in osteoblasts [15, 17]. We have previously shown that Ang II induces RANKL expression in osteoblasts via the AT1R/ERK pathway [34]. Consistently, the present study indicated that Ang II enhanced RANKL expression in RA synovial cells. Since RANKL expression in synovial cells is primarily responsible for bone erosion during joint inflammation [3], our results may provide new insights into the mechanism of bone erosion during RA.

AT1R and AT2R, the two main receptors of Ang II, are expressed in synovial tissues, and AT1R blockade effectively relieves RA symptoms [20, 22, 23, 35]. For example, Silveira et al. [22] indicated that losartan, an AT1R blocker, improved the function of joints through its potent anti-inflammatory effects in mice with antigen-induced arthritis and rats with adjuvant-induced arthritis. Moreover, an AT1R blocker was found to serve an effective RA treatment via the downregulation of the p38MAPK, ERK1/2, and NF-κB pathways [23]. In the present study, we showed that the pretreatment of synovial cells with olmesartan, an AT1R blocker, but not with PD123319, an AT2R blocker, dramatically decreased RANKL expression induced by Ang II, indicating that Ang II-induced RANKL expression was dependent on AT1R. Our results were consistent with previous reports demonstrating that Ang II-induced RANKL expression in osteoblasts is dependent on AT1R [17, 34].

Previous studies have shown that Ang II activated ERK1/2, JNK, and p38MAPK pathways in osteoblasts and embryonic stem cells via AT1R [36, 37]. In this study, we found that Ang II activated ERK1/2, JNK, and p38MAPK pathways in synovial cells via AT1R, which extended previous findings and suggested that Ang II may act through its receptors to activate the above pathways in multiple types of cells. Furthermore, previous studies have shown that the activation of ERK1/2, JNK, and/or p38 MAPK pathways induced by IL-29, IL-6, CXCL-16, and TNF-α promotes the expression of RANKL in fibroblast-like synoviocytes [12, 14, 26, 27]. We found that pretreatment of synovial cells with ERK1/2 inhibitor and JNK inhibitors, but not with a p38MAPK inhibitor, prevented Ang II-induced RANKL expression. These results indicated that the ERK1/2 and JNK pathways, but not the P38MAPK pathway, were implicated in Ang II-induced RANKL upregulation in synovial cells. Our results were consistent with a previous study indicating that P38MAPK pathway is not involved in Ang II-induced RANKL in osteoblasts [17].

Previous studies indicated that RANKL is the main cytokine implicated in Ang II-induced osteoclast formation [15, 17], and that RANKL induces osteoclast formation by binding to RANK of osteoclast precursors, thereby activating NFATC1 [28]. In the present study, we found that NFATC1 expression was higher in synovial tissues of RA patients than in those of OA patients, and that pretreatment of synovial cells with AT1R, ERK1/2, and JNK inhibitors, as well as siRNA-RANKL transfection mitigated Ang II-induced NFATC1 upregulation. These indicated that Ang II induced RANKL expression, ultimately upregulating NFATC1.

Taken together, Ang II activated the ERK1/2 and JNK pathways via AT1R, thus upregulating RANKL and NFATC1 expressions in RA synovial cells. Previous studies have demonstrated that increases of RANKL and NFATC1 expressions promote the differentiation of synovial cells into osteoclasts in synovial tissue, ultimately leading to bone destruction in RA patients [38–40]. Moreover, a line of studies have shown that Ang II promotes the formation of osteoclasts and participates in bone erosion in RA [19, 41, 42], which is consistent with our previous study [10]. Therefore, the present study may provide some novel insights of the potential mechanism that Ang II activates the ERK1/2 and JNK pathways via ATIR to promote osteoclast formation and bone destruction in RA patients, which require further elucidation.

## Conclusion

**Fig. 6:**
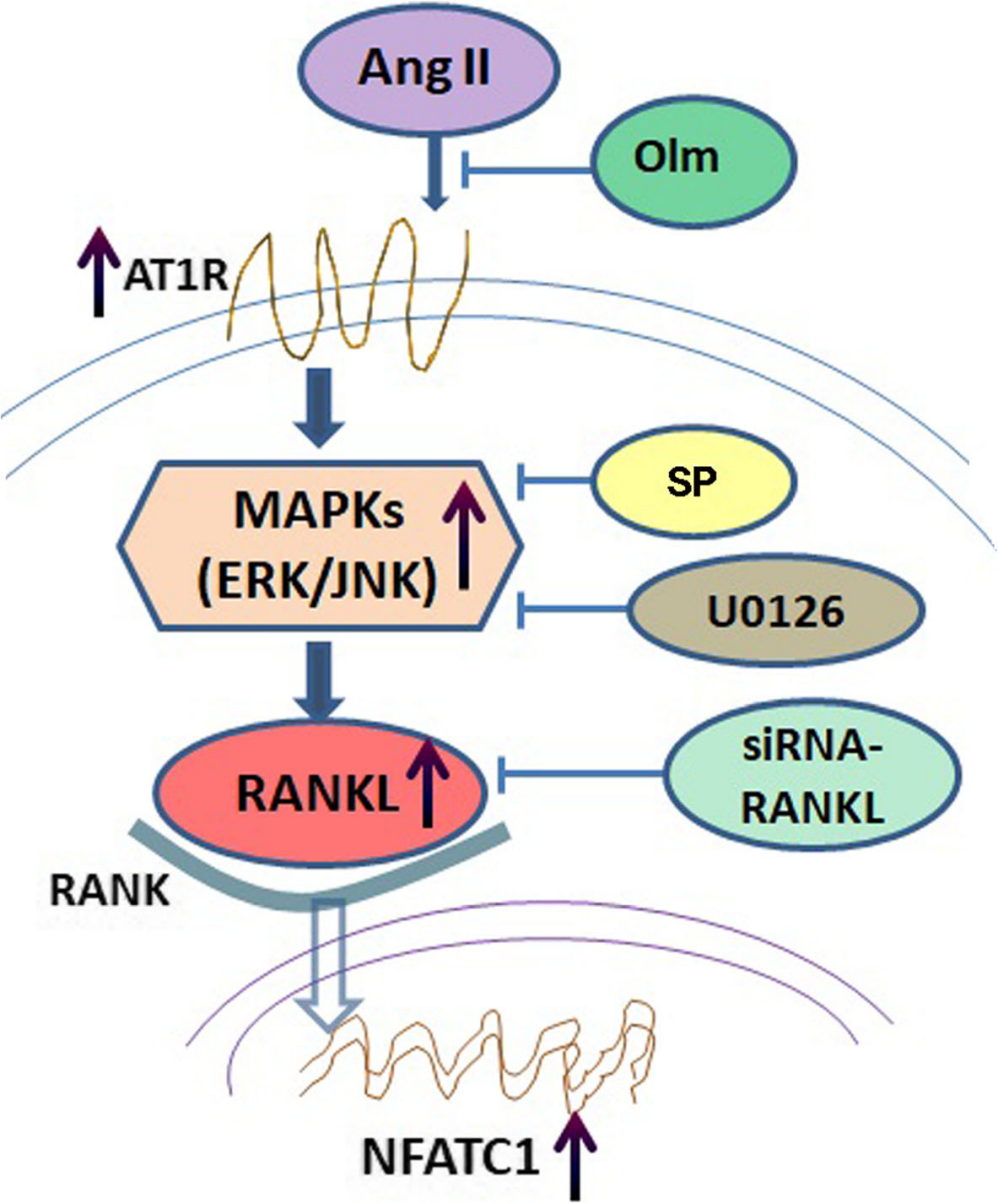
Scheme summarizing angiotensin II-induced effects on RANKL and NFATC1 expressions in synovial cells. Ang II activated the ERK1/2 and JNK pathways via ATIR, and increased RANKL expression, leading to NFATC1 upregulation in FLS

These data indicated that Ang II activated ERK1/2 and JNK pathways via ATIR, and increased RANKL expression, leading to upregulated NFATC1 expression in synovial cells (Fig. 6). These findings may provide novel insights into the role of Ang II in bone destruction in RA, and may facilitate the development of novel therapies.

## Data Availability

The datasets used in the current study are available from the corresponding author on request.

## Abbreviations

Ang II: Angiotensin II
RA: Rheumatoid arthritis
RANKL: Receptor activator of nuclear factor-κB ligand
NFATC 1: Nuclear factor of activated T cells cytoplasmic 1
AT1R: Ang II type 1 receptor
OA: Osteoarthritis
RAS: Renin-angiotensin system
ACE: Angiotensin-converting enzyme
MAPKs: Mitogen activated protein kinases
JNK: c-jun NH2-terminal kinase
ERK: Extracellular signal-regulated kinase
CXCL: CXC-motif ligand
TNF: Tumor necrosis factor
CCK8: Cell Counting Kit-8
BCA: Bicinchoninic acid
PCR: Real-time polymerase chain reaction
SiRNA: Small interfering RNA
